# Calorie restriction regulates circadian clock gene expression through BMAL1 dependent and independent mechanisms

**DOI:** 10.1038/srep25970

**Published:** 2016-05-12

**Authors:** Sonal A. Patel, Nikkhil Velingkaar, Kuldeep Makwana, Amol Chaudhari, Roman Kondratov

**Affiliations:** 1 Department of Biological, Geological, and Environmental Sciences and Center for Gene Regulation in Health and Diseases, Cleveland State University, Cleveland, OH 44115, USA.

## Abstract

Feeding behavior, metabolism and circadian clocks are interlinked. Calorie restriction (CR) is a feeding paradigm known to extend longevity. We found that CR significantly affected the rhythms in the expression of circadian clock genes in mice on the mRNA and protein levels, suggesting that CR reprograms the clocks both transcriptionally and post-transcriptionally. The effect of CR on gene expression was distinct from the effects of time-restricted feeding or fasting. Furthermore, CR affected the circadian output through up- or down-regulation of the expression of several clock-controlled transcriptional factors and the longevity candidate genes. CR-dependent effects on some clock gene expression were impaired in the liver of mice deficient for BMAL1, suggesting importance of this transcriptional factor for the transcriptional reprogramming of the clock, however, BMAL1- independent mechanisms also exist. We propose that CR recruits biological clocks as a natural mechanism of metabolic optimization under conditions of limited energy resources.

The circadian clock is an internal time-keeping system generating rhythms in physiology, metabolism and behavior^1^. In mammals, the central clock responsible for the entrainment by light resides in the brain and controls activities of multiple peripheral circadian clocks, which are located in almost every tissue of the organism^2^. Circadian clocks synchronize biochemical processes in cells, and thus may be responsible for optimization of metabolism. Disruption of the circadian clocks is associated with development of various pathological conditions, furthermore, circadian clocks have been implicated in control of aging in different organisms^3^. Disruption of the circadian clock through mutations^4^,^5^,^6^ or circadian rhythms affected by environmental interference such as shift of light/dark^7^,^8^ results in the reduced longevity. Activity of the circadian clock changes with age and it may contribute to the development of age-associated pathologies such as cancer, diabetes and neurodegeneration^9^,^10^,^11^. Calorie restriction (CR), a reduction in calorie intake without malnutrition, is a powerful intervention known for decades to slow down aging and increase lifespan across different species including mammals^12^,^13^. In the mammalian CR paradigm, the food is received in a time-restricted manner. Food is one of the strongest cues for the circadian clocks; time-restricted feeding can entrain the peripheral circadian clocks. It was proposed that the circadian clock may be a part of the mechanism facilitating the beneficial effects of calorie restriction in mammals^3^,^14^,^15^, recent data on interaction between the circadian clock and aging-controlling pathways such as the sirtuin^16^,^17^, insulin/IGF and mTOR signaling pathways^18^ support this hypothesis; however, the effect of CR on the molecular circadian clockwork has not been investigated.

At the molecular level, mammalian circadian clock is constituted of transcriptional feedback loops. The major loop involves helix-loop-helix PAS domain containing transcription factors CLOCK and BMAL1, which form a complex and regulate the transcription of other clock genes, *Periods* (*Per1* and *Per2*) and *Cryptochromes* (*Cry1* and *Cry2*), which products, in turn, inhibit CLOCK:BMAL1 transcriptional activity and their own expression, thus forming a negative feedback mechanism^19^. Another loop (called the accessory loop) consists of *Rev Erbs* (α and β) and Retinoic acid receptors (*Ror α* and *Ror γ*), that control the expression of *Bmal1* by serving as transcription repressors and activators, respectively. Additionally, the CLOCK: BMAL1 complex drives transcription of several clock-controlled transcriptional factors such as *Dec1*, *Dec2*, *Dbp*, *Hlf*, *Tef* and *E4bp4* (encoded by *Nfil3*). The clock and clock-controlled transcriptional factors generate transcriptional output of the circadian clock in metabolism and cellular biochemical processes^20^.

In this study we have assayed the effects of 30% CR on the expression of circadian clock and clock-controlled genes at the mRNA and protein level in the liver. We found that CR significantly affected circadian clockworks in a manner distinct from time-restricted feeding and fasting. We also analyzed the effects of 30% CR on clock genes expression in the liver of mice deficient for the clock transcriptional factor BMAL1. Finally, the longevity candidate genes, reported to be regulated by CR, analyzed in the circadian manner showed that the effects were gene- and time of the day-dependent.

## Results

### Effect of CR on core clock genes expression

30% CR is one of the most commonly used CR for C57B6 mice^21^,^22^, therefore, we selected this feeding paradigm to perform the gene expression analysis. To assay the effects of CR on the molecular circadian clockworks, we compared expression of the core circadian genes across the day in the liver of mice that were subjected to different feeding paradigms. *Ad libitum* (AL) fed mice had unlimited access to food throughout the day. CR mice received 30% less of their daily AL food intake for two months. In this group food was provided two hours after the light was turned off (ZT14); indeed, mice are nocturnal animals, so the nighttime feeding is most physiologically relevant. In the third, time restricted (TR) feeding group, animals were provided with the 100% of their average daily AL food intake at ZT14 (the same time as the CR group) for two weeks; this time period is sufficient to reset circadian clocks in peripheral tissues^23^,^24^. We noticed that animals in both CR and TR groups consumed all the food within the first 3–5 hours, in agreement with our previous report on TR feeding^25^; therefore, the TR group represents an appropriate control for the CR group. Indeed, animals consume the food at the same time as the CR group, but there is no reduction in the amount of consumed calories. The fourth group of animals has also been on the TR regimen for two weeks, but the animals did not receive food on the day of tissue collection. Thus, this fasting (F) group experiences food deprivation for 24 to 48 hours, and thus represents another control for the CR group: the control for sharp reduction in the calorie intake without any metabolic adaptation, in contrast to CR.

**Figure 1:**
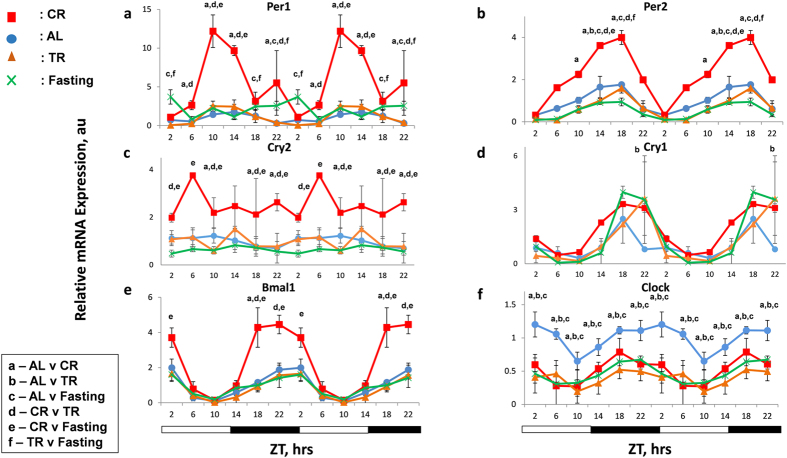
Effect of CR on rhythms of circadian clock genes. mRNA expression of core clock genes – (**a**) *Per1*, (**b**) *Per2*, (**c**) *Cry2,*(**d**) *Cry1,* (**e**) *Bmal1 and* (**f**) *Clock -* was assayed in the liver of mice (n = 3 per time point) subjected to the following feeding regimens: ad libitum (AL) – blue circles, solid line; 30% calorie restriction (CR) – red squares and solid lines; time restricted feeding (TR)– orange triangles and solid lines, fasting (F) – green cross and solid lines . For convenience all data are double plotted. Data represents mean ± SD; statistically significant (p < 0.05) effects of the feeding (analyzed by two ways ANOVA) at a given time point are indicated by: (**a**)**-** between AL and CR groups, (**b**) – AL and TR, **c**- AL and fasting, (**d**)**-** CR and TR, (**e**) – CR and Fasting, (**f**)- TR and Fasting, Light and dark bars at the bottom represent light and dark phase of the day. ZT0 is the time when light is on and ZT12 is the time when light is off. Food for CR and TR groups was provided at ZT14.

As expected, the expression of core clock genes exhibited oscillatory pattern of expression in the liver of AL mice (Fig. 1) in agreement with previously reported data. We observed that 30% CR dramatically affected the expression of circadian clock genes. According to the two way ANOVA analysis, mRNA expression for *Bmal1*, *Per1*, *Per2, Cry2,* (Fig. 1) and *Rev Erb β*, *Per3* and *Ror γ* (Supplementary Fig. S1) were significantly affected by CR. Cosinor wave analysis of circadian rhythms in the expression of the circadian clock genes revealed that most feeding regimens did not significantly affect rhythmicity or acrophase for most of the genes, however, CR and fasting disrupted circadian rhythms in the expression of *Per1* and CR induced circadian rhythms in the expression of *Clock* gene. CR did not affect *Cry1, Rev Erb α* and *Ror α* mRNA expression (Fig.1 and Supplementary Fig. S1) and even statistically significantly reduced the expression of the *Clock* gene. TR had a similar effect as CR on the expression of *Rev Erb β* and *Clock* genes. Thus, for Per1, Per2, Per3, Bmal1 and Cry2 genes the effect on the expression was CR specific and different from the effects of TR and Fasting.

### Effect of CR on clock-controlled genes expression

**Figure 2:**
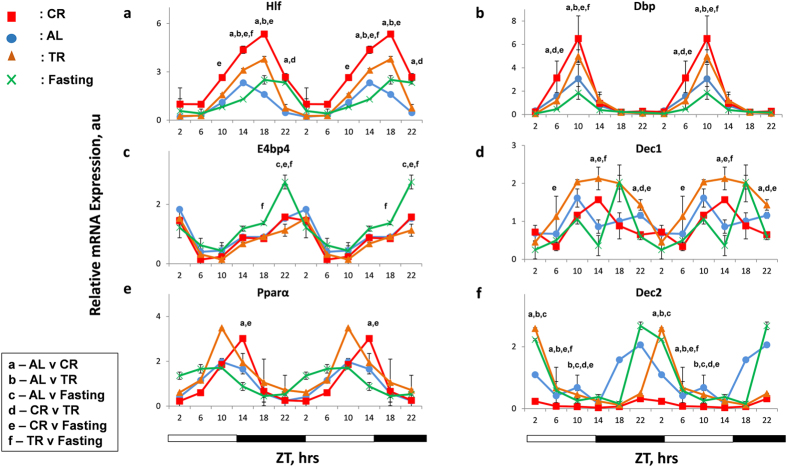
CR effects on rhythms of clock controlled genes expression. mRNA levels of several clock controlled genes – (**a**) *Hlf*, (**b**) *Dbp*, (**c**) *E4bp4,* (**d**) *Dec1,* (**e**) *Pparα and* (**f**) *Dec2* was assayed in the liver of mice (n = 3 per time point) subjected to the following feeding regimens: ad libitum (AL) –blue circles, solid line; 30% calorie restriction (CR) – red squares and solid lines; time restricted feeding (TR) orange triangles and solid lines and fasting (F) – green cross and solid lines. For convenience all data are double-plotted. Data represents mean ± SD; statistically significant (p < 0.05) effects of the feeding (analyzed by two ways ANOVA) at a given time point are indicated by: (**a**)**-** between AL and CR groups, (**b**) – AL and TR, **c**- AL and Fasting, (**d**)**-** CR and TR, (**e**) – CR and Fasting, (**f**)- TR and Fasting, Light and dark bars at the bottom represent light and dark phase of the day. ZT0 is the time when light is on and ZT12 is the time when light is off. Food for CR and TR groups was provided at ZT14.

Several clock-controlled transcription factors, such as *Hlf, Tef, Dbp, E4bp4, Dec1* and *Dec2*, have been shown to be the transcriptional targets of CLOCK/BMAL1 complex, and reported to oscillate robustly with different phases in the liver^26^,^27^. Previous reports have shown feeding/fasting cycle to have variable effects on the mRNA expression of some of these transcription factors^28^,^29^. We assayed the mRNA expression of these clock-controlled genes in the liver of 30% CR, TR, Fasting and AL mice by qPCR. CR significantly induced the expression of *Hlf* and *Tef* (Fig. 2a, Supplementary Fig. S1f) at several time points; TR also affected the expression of these genes. CR up regulated the expression of the D box transcription factor (Dbp,) at ZT6 and ZT10, whereas Fasting reduced *Dbp* expression (Fig. 2b), in agreement with earlier report in fasting animals^30^. *E4bp4* mRNA levels were not affected by CR or TR, but were up regulated by Fasting later at night time points compared to AL (Fig. 2c). There was statistically significant reduction in *Dec1* expression at ZT22 in CR, it was increased in TR group and, as previously demonstrated, it was down regulated at several time points during the day in the Fasting group (Fig. 2d). *Dec2* mRNA expression was significantly down regulated in CR animals and it was upregulated in TR and Fasting at several time points (Fig. 2f). Variable results have been observed in *Pparα* mRNA expression upon CR^31^,^32^. We showed that the effect of 30%CR on *Pparα* mRNA expression was not statistically significant (Fig. 2e). These results indicate that CR affects the expression of clock-controlled transcription factors; the effect of CR on Dbp, Dec1 and Dec2 is statistically significantly different from TR or Fasting.

### CR leads to reduced levels of CRY1 protein

**Figure 3:**
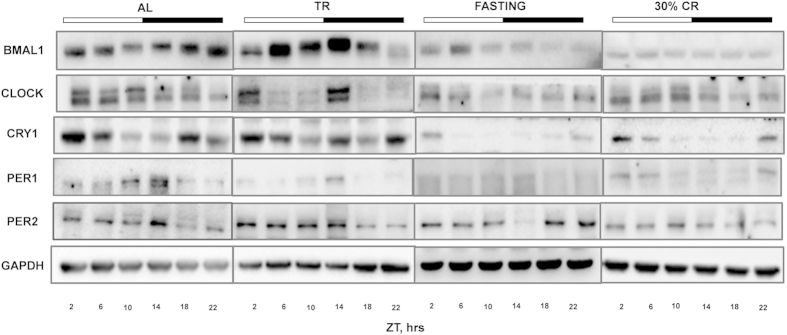
Effect CR on rhythms in clock protein levels. Representative Western blots for clock proteins; BMAL1, CLOCK, CRY1, PER1 and PER2 assayed in the livers of mice (liver samples from three mice were pooled together at each time point) subjected to the following feeding regimens: AL – ad libitum, CR – 30% calorie restriction, TR –time-restricted feeding, F – fasting. Light and dark bars on the top of the figure represent light and dark phase of the day. ZT0 is the time when light is on and ZT12 is the time when light is off. Food for CR and TR groups was provided at ZT14, F group received food at ZT14 for two weeks and did not receive the food at the day of tissue harvesting.

Next we assayed the protein levels of the core clock genes (BMAL1, CLOCK, PER1, PER2 and CRY1) in the liver of mice from different feeding groups (30% CR, TR, Fasting and AL). Rhythmic oscillations at the protein level in our AL group were similar to that demonstrated in previous reports^33^,^34^. BMAL1, PER1 and PER2 have shown circadian rhythms, while daily changes in CLOCK and CRY1 were not circadian according to the cosinor wave analysis^33^,^34^. Based on the two way ANOVA, CR significantly reduced the protein level of core clock gene, CRY1, while the expression of CLOCK, BMAL1, PER1 and PER2 were downregulated but the effect was not statistically significant as shown in Fig. 3. CLOCK protein levels was down regulated at one time point upon Fasting (Fig. 3 and Supplementary Fig. S2). PER1 and PER2 showed temporal changes in AL and TR as expected, having a peak around ZT 14, whereas in Fasting maximum abundance was around ZT 22-2 for Per2 and around ZT 2 for Per1. The CRY1 protein level was dramatically reduced in both CR and Fasting groups compared to AL and TR (Fig. 3 and Supplementary Fig. S2) at all tested time points. Thus, both Fasting and CR led to significant down regulation of CRY1 on protein level at every time point, at the same time the effect of Fasting on the expression of CLOCK was time of the day dependent. TR effect on the expression for all tested clock genes was not statistically different from AL.

### BMAL1 is involved in CR effects on clock genes expression

**Figure 4:**
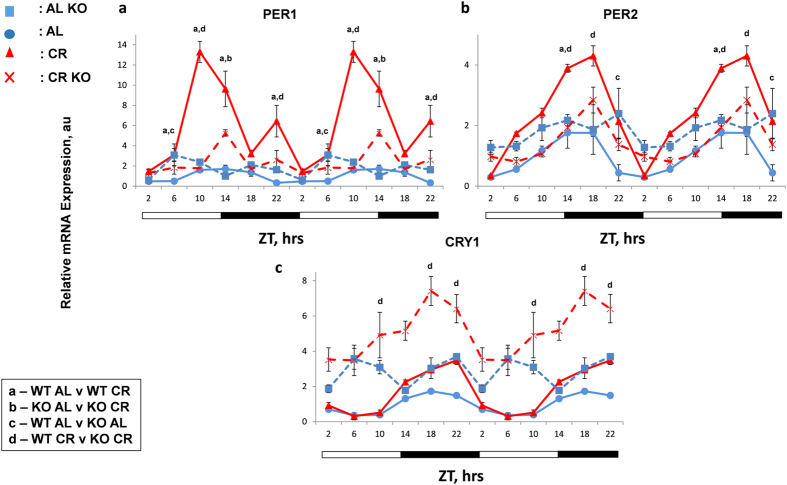
BMAL1 dependent and independent regulation of circadian clock gene expression by CR. Circadian patterns of mRNA expression of clock genes – (**a**) *Per1,* (**b**) *Per2* and (**c**) *Cry1,* were assayed by qPCR in the liver collected at 4 hours interval over period of 24 h from wild type (WT) and *Bmal1*−/− (KO) mice (n = 3 per time point) subjected to the AL and CR: WT AL controls-blue circles and solid lines; WT CR- red triangles and solid lines; KO AL –blue squares, dashed lines; KO CR- red cross and dashed lines. All the graphs are double-plotted. Data represents mean ± SD; statistically significant (p < 0.05) differences between the genotypes and effects of different diets across the time (analyzed by three way ANOVA) are indicated by: (**a**)**-** between WT AL and WT CR groups, (**b**) – KO AL and KO CR, **c**- WT AL and KO AL, (**d**)**-** WT CR and KO CR. Light and dark bars at the bottom represent light and dark phase of the day. ZT0 is the time when light is on and ZT12 is the time when light is off. Food for CR was provided at ZT14.

Because CR had a significant effect on clock genes mRNA expression and protein levels, our next question was if the effect of CR was through the regulation of BMAL1 transcription activity. We analyzed the mRNA expression of clock genes in whole body *Bmal1*−/− mice subjected to 30% CR for 2 months. Similar to others, our results also demonstrated that expressions of *Per1*, *Per2* and *Cry1* were arrhythmic in AL fed *Bmal1*−/− mice (Fig. 4 and cosinor analysis). According to three way ANOVA, the effect of CR was statistically significant for all three tested genes in wild type but in *Bmal1*−/− mice the effect of CR was significant only for *Cry1*. Thus, BMAL1 is required for the effect of CR on *Per1* and *Per2* but not for *Cry1* mRNA expression.

### Effects of feeding regimens on expression of longevity candidate genes

**Figure 5:**
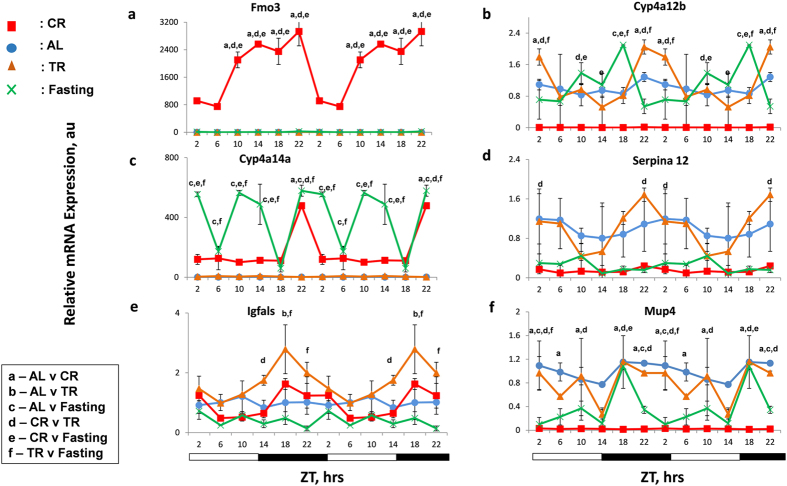
CR has time- and gene-specific effect on longevity associated candidate gene expression. mRNA levels of several longevity associated candidate genes – (**a**) *Fmo3*, (**b**) *Cyp4a12b*, (**c**) *Cyp4a14a,*(**d**) *Serpina12,* (**e**) *Igfals and* (**f**) *Mup4* in the liver of mice (n = 3 per time point) subjected to the following feeding regimens: ad libitum (AL) – blue circles, solid line; 30% calorie restriction (CR) – red squares and solid lines; time restricted feeding (TR)– orange triangles and solid lines, fasting (F) – green cross and solid lines. For convenience all data are double-plotted. Data represents mean ± SD; statistically significant (p < 0.05) effects of the feeding (analyzed by two ways ANOVA) at a given time point are indicated by: (**a**)**-** between AL and CR groups, (**b**) – AL and TR, (**c**)- AL and Fasting, (**d**)**-** CR and TR, (**e**) – CR and Fasting, (**f**)- TR and Fasting. Light and dark bars at the bottom represent light and dark phase of the day. ZT0 is the time when light is on and ZT12 is the time when light is off. Food for CR and TR groups was provided at ZT14.

Effects of CR on the expression of *Per1* and *Per2* were previously reported: *Per1* and *Per2* genes were identified, using multiple microarray data analysis, among the group of genes with differential expression in the multiple tissues such as liver, brain, skeletal muscles of mice subjected to lifespan extending diets such as CR^35^. These genes were proposed as longevity-associated candidate genes and later for some of them their differential expressions were confirmed in tissues of long lived dwarf mice^35^. However, one important caveat in these studies is that the time of tissue collection was not defined, making the comparison between samples complicated. Indeed, microarray analysis of the expression upon CR was performed in multiple groups independently and for every selected gene, including *Per1* and *Per2*, the effects of CR on the expression was not confirmed in every study^35^,^36^. We hypothesized that the observed discrepancy in results between different studies is due to different time of sample collections and the expression of longevity candidates genes might significantly change across the day. Indeed the effects of CR on *Per1* and *Per2* expressions in our study were significant at some time and not significant at another time of the day (Fig. 1). We selected ten longevity candidate genes: the expressions of Fmo3, *Cyp4a14, Parp16 and Igfals* genes were reported to be up regulated upon CR while the expressions of *Cyp4a12b, Mup4, Hes6*, *Hsd3β5, Serpina12* and *Alas2* were reported to be down regulated upon CR. We assayed the mRNA expression of these genes across different time of the day in the liver of mice maintained on different diets. Interestingly, some of the genes tested such as *Hsd3β5 and Serpina12* exhibited rhythmic expression around the day. We did not find statistically significant effect of CR on the expressions of *Igfals* and *Serpina12.* The expressions of 8 candidate genes was statistically significant either up or down regulated upon 30% CR (Fig. 5 and Supplementary Fig. S3) at least at some time points, in agreement with published microarray data; however, only for a subset of longevity candidate genes the effect was CR-specific. Expression of flavin monoxygenase (*Fmo3*) (Fig. 5a) was up regulated significantly with high amplitude rhythms upon CR, but not upon TR or Fasting. Expression of *Cyp450* gene, *Cyp4a12b,* (Fig. 5b) was down regulated upon CR, while there was no effect of TR or Fasting. Expression of *Parp16* (Supplementary Fig. S3a) was statistically significantly up regulated, and expression of, *Mup4* and *Alas2* (Fig. 5f and Supplementary Fig. S3c) was statistically significantly down regulated upon CR at several time points, while at other time points there was no difference between CR and AL, TR or Fasting samples. Effects on the expression of other tested target genes were not CR-specific: for example, while the expression of *Cyp450 gene, Cyp4a14* (Fig. 5c) was dramatically up regulated upon CR, and expressions of *Hes6* (Fig. S3b) and *Hsd3β5* (Supplementary Fig. S3d) were down regulated upon CR (in agreement with the published microarray data), similar up or down regulations were detected upon Fasting too. These results emphasize the importance of the circadian approach for the study of the effect of CR on transcriptome. The effect of CR on Fmo3, Cyp4a14a, Cyp4a12b, Mup4, Hes6, Parp16, and Hsd3B5 was statistically significantly different from effect of TR and Fasting.

## Discussion

The calorie restriction leads to multiple physiological and metabolic changes, which may contribute to longevity^12^,^13^. Two major features of the CR paradigm in mammals are (1) reduced calorie intake and (2) periodicity in availability of food. This periodic feeding resembles another feeding paradigm, known as time-restricted feeding. It was reported that TR could affect the circadian clocks^3^. The expressions of circadian clock genes in the liver can be affected by feeding time: for example, restricting the time of food availability to the light phase of the day (non-physiological for rodents) leads to a shift in the expression phase of the clock genes in the liver compared with AL fed mice, which predominantly feed during the dark phase of the day^24^,^37^,^38^. The composition of food also has a significant effect on gene expression: the high fat diet results in reduced amplitude of the expression for most clock genes in the liver^39^, and time-restricted feeding applied for the high fat diet restores the rhythms in the expression^39^. Interestingly, the expression of clock genes is also affected by pathological conditions: for example, development of insulin resistance and diabetes in streptozotocin-treated mice is accompanied by a significant induction of expression of several clock genes (*Per1* and *Per2*, *Bmal1* and *Cry1*)^40^ . Detailed molecular mechanisms connecting feeding and nutrients with clock gene expression need to be studied; nutrient or energy status-responding chromatin modifying enzymes interacting with clock machinery have been proposed as important mediators^41^.

In the present study we compared short-term 30% CR with TR and fasting. Food was provided at the same time for all groups during the dark phase of the day (the time of normal feeding for AL group); as expected, the phase of clock gene expression was not significantly affected by CR or TR. CR significantly induced the expression of several clock and clock-controlled genes. Circadian clocks in tissues are formed by individual cellular oscillators; thus, one possible explanation of the observed effect of CR is robust synchronization of these individual cellular oscillators. However, upon synchronization one must expect increase in the amplitude of the rhythms rather than the effect on average expression. We found that CR affected the daily average in the expression of *Per1, Per2, Cry2, Dec2* and *Hlf* genes, suggesting that increased synchronization cannot be the only mechanism. In addition, expression of some clock genes such as *Rev-Erb α* was not affected by CR. Finally, CR-induced changes in gene expression were different from those of TR, suggesting that periodic feeding is not the major contributor to the effects of CR on clock genes expression. In the study by Mendoza *et al*., effect of CR on the expression of circadian clock genes has been assayed in the SCN^42^. While we cannot compare the results of this study with our results directly because different tissues have been assayed and the food was provided at different times of the day (at ZT6 in Mendoza *et al*. and at ZT14 at our study), in both studies CR has significant effect on the expression of several but not all tested clock genes. Interestingly, analysis of published microarray data on the influence of CR on mRNA expression in different tissues in mice pooled from multiple independent studies performed by different groups^43^,^44^ identified two clock genes, *Per1* and *Per2,* among several genes whose expression was affected upon CR in many tissues. However, the up regulation of *Per1* and *Per2* expression was not detected in every study, for example, up regulation of *Per1* and *Per2* mRNA expression in the liver was detected in two out of seven studies^43^. Our data confirmed that the expressions of *Pers* are indeed affected by CR and provided a potential explanation of the discrepancy between results published by different groups, as different groups may utilize different time points for tissue collection.

Sharp reduction in calorie intake also affects expression of several clock genes: *Per1* expression is up regulated, and *Per2* expression is down regulated upon fasting^28^,^30^. In our study fasting also led to an increase in *Per1* expression; this change was similar to that one induced by the CR; however, the magnitude of the effect was modest compared to CR. In sharp contrast to the effects of CR, which led to increased expression of *Per2, Per3, Cry2* and *Ror γ* mRNAs, fasting resulted in decreased expression of these genes. Finally, fasting did not affect expression of *Bmal1* (induced by CR) and *Dec2* (suppressed by CR). Our data argue that CR results in the changes in the molecular circadian clockwork in the liver; these changes are, most likely, a result of metabolic adaptation to CR, because they are different from the effects of periodic feeding and sharp reduction in calorie intake.

Expression of circadian clock genes is controlled by several transcriptional factors. The BMAL1/CLOCK (BMAL1/NPAS2) complex is considered as a major regulator of *Per1, Per2, Cry1, Cry2, Revs, Rors, Decs, Ppars* and *Dbp* gene expression. The effect of CR on expressions of some of these genes was significantly impaired in the liver of *Bmal1*−/− mice, which supports the involvement of BMAL1-containing transcriptional complexes in the observed changes in the expression. On the other hand, CR affected not all of the BMAL1 targets; moreover, the induction of *Cry1* expression by CR was intact in *Bmal1*−/− mice, suggesting existence of both BMAL1-dependent and BMAL1-independent mechanisms. The existence of BMAL1-independent mechanisms is not surprising, indeed, it was reported that circadian food anticipation rhythms are BMAL1-independent^45^,^46^. The circadian clockwork is organized as a network of many positive and negative feedback loops^2^: for example, E4BP4 contributes to the circadian control of the expression of *Per2, Cry1, Clock* and *Ror γ* genes, and transcriptional factors, REV-ERB and ROR family control the expression of *Bmal1* and *E4bp4*^27^. Furthermore, clock gene expression is regulated on posttranscriptional and posttranslational levels^47^, therefore, the effect of CR on the expression may be complex, and cannot be explained exclusively through the activation of only one transcriptional factor.

Effects of different feeding regimens on the expression of clock genes on the protein level are much less studied. In contrast to increased expression of several clock genes on the mRNA level, we detected statistically significant down regulation of CRY1 and tendency towards reduction for other genes (the difference has not reached statistical significance) upon 30% CR. This discrepancy implies a regulation at the post-transcriptional level, in agreement with that, we observed reduced levels of BMAL1 and CLOCK proteins and increased mRNA levels of their transcriptional targets. CRY1 is a suppressor of CLOCK/BMAL1 transcriptional activity. We found that CR led to dramatic reduction of the CRY1 protein level, which supports an increase in mRNA expression of BMAL1/CLOCK targets. Fasting had a similar effect as CR on the CRY1 protein level. Interestingly, in cells depleted of glucose, AMPK phosphorylates CRY1, which leads to CRY1 degradation^48^. Under both CR and fasting conditions, the blood glucose level is significantly reduced^49^,^50^. Hence, it is possible that AMPK plays a role in the observed reduction of the CRY1 protein level. At the same time, reduction in the blood glucose level upon CR or fasting is relatively moderate (in comparison with severe glucose depletion in cell culture), and it is currently a matter of debate whether it is sufficient to activate AMPK *in vivo*^51^. Therefore, other mechanisms such as reduced CRY1 translation or degradation can be involved^52^,^53^. It is also necessary to mention that while CR and fasting have similar effects on CRY1 protein level, currently we cannot say if the down regulation of CRY1 protein upon CR and fasting occur through the same molecular mechanism or through different mechanisms. We also did not detect a direct correlation between *Per1* and *Per2* expression on the mRNA and protein levels. It is known that stability of PERs is regulated by phosphorylation and formation of complex with CRYs; thus, one may expect that a reduced level of CRY1 is associated with an increased mRNA and decreased protein levels of *Pers*. Importantly, regardless of the exact mechanisms affecting circadian protein levels, fasting and CR have very different effects on the circadian mRNA expression, in contrast to the effects of CR, which affects the mRNA expression of multiple circadian clock and clock controlled genes, fasting did not affect mRNA expression of the same gene significantly. Therefore, while reduced level of CRY1 protein may contribute to the increased expression of clock genes, absence of the effect upon fasting argues for existence of CRY-independent mechanisms also.

Mechanisms of CR are not well understood; several dozens of genes were put forward as targets and mediators of CR through the transcriptome analysis and called the candidate longevity genes. However, in most of these studies the expression analysis was performed at only one time point. We assayed circadian profiles of the expression of ten candidate longevity genes: selected genes known to influence different processes such as xenobiotic metabolism, protein binding and transport, steroid biosynthesis, heme binding and cell proliferation^35^. Although their importance in aging and lifespan extension has not been directly established, some of these genes could be promising candidates required for longevity. We found that two out of ten genes passed the criteria to be regulated exclusively by CR at all the time points tested: expression of *Cyp4a12b* was suppressed and expression of *Fmo3* was induced by CR but not by TR or fasting. Four genes (*Parp16*, *Alas2*, *Igfals* and *Mup4*) were also regulated by CR, but the effect on mRNA expression was time of the day-dependent. This time-of-the-day dependence of CR may explain the contradictory results for some CR-regulated genes: for example, *Parp16*^36^, which according to our data is significantly up regulated only at ZT2, 10 and 14 but not at ZT6, 18 and 22. Importantly, the expression of 7 out of 10 tested genes have shown changes across the day and 2 out them have shown circadian rhythms in the expression. Analysis of gene expression in circadian manner is still not a common practice in many fields but our data argue that analysis of the circadian rhythms must be taken into account for future studies. Both CR and fasting up or down regulated four candidate longevity genes; therefore, we conclude that regulation of these genes is not CR-specific. These results argue for taking data accumulated in different studies for the analysis of CR transcriptome with great caution. Interestingly, 6 out of 11 tested circadian clock genes, and 6 out of the 10 tested longevity-associated candidate genes were regulated by CR, suggesting that clock genes may be considered as longevity-associated candidate genes. Indeed, analysis of longevity among different species recognized circadian rhythms as one of the candidate target contributing towards the evolution of longevity^54^ further strengthening our hypothesis.

CR and TR have different effects on circadian clocks: in contrast to TR, which does not affect the clock in the SCN, CR can entrain the SCN clock^55^. Metabolic benefits of TR were demonstrated for rodents fed on the high fat diet^39^; however, metabolic benefits of TR for animals on the regular chow have not been assayed. While our data cannot exclude a potentially beneficial impact of TR, there is a clear difference between CR and TR in terms of expression of circadian clock, clock-controlled and longevity candidate genes. Interestingly, in contrast to CR, TR does not extend lifespan in rodents; therefore, beneficial effects of CR on longevity correlate with the effects of CR on the circadian clocks. CR results in significant metabolic changes in mammals and other organisms^13^, the circadian clocks are major regulators of metabolism^56^, and according to our data, CR significantly affected circadian clockworks, including the expression of the core circadian clock genes and circadian clock controlled transcriptional factors, which provide circadian output in metabolism. One possible interpretation if the results is that the CR disrupts the circadian molecular clockwork, because the expression of many clock genes has been significantly changed. This is to some extent paradoxical because clock disruption is associated with many pathological conditions and was proposed as a contributing factor to the diseases. However, CR has beneficial effects on the longevity in spite of clock disruption. Alternatively, we propose that CR-dependent effect on the circadian clocks is a necessary component of the metabolic adaptation to CR. Indeed, maintenance of physiological homeostasis under conditions of limited energy supply requires essential optimization of biochemical processes. According to the existing paradigm, the circadian clocks synchronize metabolic processes through the control of expression of multiple rate-limiting enzymes as a result of regulation of circadian clock output transcriptional factors^57^. CR recruits circadian clock-dependent mechanisms for optimization in order to increase fitness of the organism. Future study focused on the effects of CR in circadian clock mutants will help to clarify connections between clock and CR.

## Materials and Methods

### Ethics Statement

All the animal studies were performed with approval from the Institutional Animal Care and Use Committee (IACUC) of Cleveland State University (Protocol No. 21124-KON-S). The care and use of mice were carried out in accordance with the guidelines of the Institutional Animal Care and Use Committee (IACUC) of the Cleveland State University.

### Experimental animals

Wild type and whole body *Bmal1*−/− male mice were used for the experiments. Mice were of C57B6J background. *Bmal1*−/− mice were obtained from laboratory of Dr. Bradfield^58^. Animals were maintained on the 12:12 light:dark cycle with lights on at 7:00 am, and fed the 18% protein rodent diet (Harlan). The *ad libitum* (AL) group had unrestricted access to food. Calorie restriction (CR) was started at 3 months of age. For the first week animals had been on 10% restriction, for the second week on 20% restriction and on 30% restriction for the rest of the experiment. The CR group received their food once per day at ZT14 (two hours after lights were off). After two months of CR, tissues were collected at six different time points across the day. The time restricted (TR) feeding group started to receive 100% of their average daily intake as one meal at ZT14. TR started at 4.5 months of age, mice were on TR for 2 weeks before tissue collection. The fasting group (F) was on the same TR feeding regime for 2 weeks, but did not receive food on the day of tissue collection. All groups had unrestricted access to water. All tissue collection experiments were performed on 5 months old wild type (WT) and *Bmal1*−/− male mice. For all experiments three animals of each genotype, feeding regimen and time point were used.

### RNA isolation and analysis of mRNA expression

For gene expression studies, liver tissues from 3 male mice on each diet (AL, CR, TR and Fasting) and for both genotype (WT and *Bmal1*−/−) were collected every four hours throughout the day, and stored at −80 °C. Total RNA was isolated using TriZol reagent (Invitrogen, Carlsbad, CA) as per the manufacturer’s protocol. Briefly, frozen liver piece was minced in 1 ml TriZol reagent with pestle on ice. Following chloroform extraction step, total RNA was precipitated with isopropanol by centrifugation and pellet obtained was washed with 70% Ethanol. RNA pellet was diluted in 30 μl of RNAse-free water and quantified on Nanodrop. RNA integrity was checked on 1% agarose gel run at 90 V for 30 minutes. 20 μl of RT mix was prepared using 1 μg of RNA, 50 ng of 50 uM random hexamer (N8080127, Invitrogen), 10 mM dNTP (DD0058, Biobasic), 0.1 M DTT and RNaseOUT™ Recombinant RNase Inhibitor (10777-019, 40 units/μl). It was then reverse transcribed by qPCR machine using 200 u/μl of SuperScript^®^ III Reverse Transcriptase (18080-044, Invitrogen) as per the manufacturer’s instructions. Incubation conditions used were: 65 °C for 10 minutes followed by incubation on ice for 1 minute; 25 °C for 5 minutes; 50 °C for 60 minutes; Inactivate the reaction by heating at 70 °C for 15 minutes. RNA quantification was performed using qPCR with Universal Syber Green mix (1725125, BioRad). The reaction was carried out in triplicates for the gene of interest and in duplicates for the normalizing control using CFX96 qPCR Detection System (BioRad) with 50 ng of cDNA. Thermal cycling conditions used were according to the instructions of SYBR Green mix protocol and are briefly described in and relative mRNA abundance was calculated using the comparative delta-Ct method with ribosomal 18S rRNA and Gapdh as reference genes as described in^25^. Water was used as the negative control for the qPCR analysis. Product specificity was confirmed by melting curve analysis while primer pair efficiency was calculated by generating standard curve using serial dilutions of standard. Primers used for the analysis of expression are listed in Supplementary materials.

### Immunoblot analysis

For analysis of protein expression tissues from three male mice per time point were used for each feeding regimens and both genotypes (WT and *Bmal1*−/−). Figure 3 represents Western blotting when three liver samples from three different mice were pooled together at each time point for each diet. For quantitative data presented in Supplementary Fig. S3, liver samples from individual mice (N = 3) were run to estimate a variability between biological replicates and calculate means and errors. For lysates preparation, frozen liver pieces were lysed in cell signaling lysis buffer with Protease/Phosphatase Inhibitor Cocktail (Cell Signaling Technology, Beverly, MA, USA) using sonicator. Protein concentration was determined by Bradford protein assay kit according to manufacturer’s protocol using spectrophotometer and lysates were stored at −80C. 45 ug of protein was loaded on 3–8% tris-acetate and 4–12% bis-tris gels (Invitrogen). Protein was transferred on PVDF membrane at 110 mAmp. Equal loading of proteins was checked by Ponceau stain. Primary antibodies anti-CRY1 (SAB biosciences), anti-BMAL1 (Santacruz Biotechnology), anti-CLOCK (kindly provided by Dr Marina Antoch, Roswell Park Cancer Institute), anti-PER2 (Alpha Diagnostics), anti-PER1 (Thermoscientific) and anti-GAPDH (Cell Signaling) were used for Immunoblot analyses. Protein analysis and quantification was done using Scientific Imaging film and Odyssey FC imaging system (LI-COR).

### Statistical analysis

For each feeding type and for each genotype, at least three animals for every time point were used for all experiments. Data are shown as average +/− S.D. To assay the effect of feeding and time of the day on mRNA and protein level we performed two-way ANOVA. If the effect of feeding and/or time of the day was found to be statistically significant, Bonferroni correction was used to calculate p value for pairwise comparison between each feeding regimen at every time of the day. To assay the effect of genotype, feeding and time of the day on mRNA levels first analysis was performed using three-way ANOVA, If the effect of feeding, genotype and/or time of the day was found to be statistically significant, Bonferroni correction was used to calculate p value for pairwise comparison between each feeding regimen at every time of the day. IBM SPSS Statistics 20 and GraphPad Prism Version 5.04 software packages were used for statistical analysis. P < 0.05 was considered as statistically significant difference.

## Additional Information

**How to cite this article**: Patel, S. A. *et al*. Calorie restriction regulates circadian clock gene expression through BMAL1 dependent and independent mechanisms. *Sci. Rep.* **6**, 25970; doi: 10.1038/srep25970 (2016).

## Supplementary Material

Supplementary Information
