# Supplementary material for: Calorie restriction regulates circadian clock gene expression through BMAL1 dependent and independent mechanisms
Source: Sci Rep. 2016 May 12;6:25970. doi: 10.1038/srep25970 (PMC4864379; doi:10.1038/srep25970)
Supplement: Supplementary Information [file srep25970-s1.docx]

**Calorie restriction regulates circadian clock gene expression through BMAL1 dependent and Independent mechanisms**

Sonal A Patel, Nikkhil Velingkaar, Kuldeep Makwana, Amol Chaudhari and Roman Kondratov*

Department of Biological, Geological, and Environmental Sciences and Center for Gene Regulation in Health and Diseases, Cleveland State University, Cleveland, OH 44115.

* - corresponding author: Tel: 216-523-7199; e-mail: [r.kondratov@csuohio.edu](mailto:r.kondratov@csuohio.edu)

**Supplementary Material**

**Supplementary Table S1. Primer sequences of clock and clock controlled genes for qPCR.**

**Supplementary Table S2. Primer sequences of Longevity-associated genes for qPCR.**

**Supplementary Fig. S1. CR activates BMAL1/CLOCK transcriptional targets**

mRNA expression BMAL1 core target genes – (a) *Per3*, (b) *Rev Erb α*, (c) *Ror α*,(d) *Rev Erb β*, (e) *Ror γ* and (f) *Tef* *-* was assayed in the liver of mice (n=3 per time point) subjected to the following feeding regimens: ad libitum (AL) – blue circles, solid line; 30% calorie restriction (CR) – red squares and solid lines; time restricted feeding (TR)– orange triangles and solid lines, Fasting (F) – green cross and solid lines . For convenience all data are double plotted. Data represents mean ± SD; statistically significant (p<0.05) effects of the feeding (analyzed by two ways ANOVA) at a given time point are indicated by: **a-** between AL and CR groups, **b** – AL and TR, **c**- AL and Fasting, **d-** CR and TR, **e** – CR and Fasting, **f**- TR and Fasting. Light and dark bars at the bottom represent light and dark phase of the day. ZT0 is the time when light is on and ZT12 is the time when light is off. Food for CR and TR groups was provided at ZT14.

**Supplementary Fig. S2. Quantification analysis of protein expression of core clock genes in liver**

Protein levels of (a) BMAL1, (b) PER1, (c) CLOCK, (d) CRY1 and (e) PER2 proteins in the liver of mice (n=3 per time point) subjected to the following feeding regimens: ad libitum (AL) – blue circles, solid line; 30% calorie restriction (CR) – red squares and solid lines; time restricted feeding (TR) – orange triangles and solid lines, Fasting (F) – green cross and solid lines. For convenience all data are double plotted. Data represents mean ± SD; statistically significant (p<0.05) effects of the feeding (analyzed by two ways ANOVA) at a given time point are indicated by: **a-** between AL and CR groups, **b** – AL and TR, **c**- AL and Fasting, **d-** CR and TR, **e** – CR and Fasting, **f**- TR and Fasting. Light and dark bars at the bottom represent light and dark phase of the day. ZT0 is the time when light is on and ZT12 is the time when light is off. Food for CR and TR groups was provided at ZT14.

**Supplementary Fig. S3. CR has time- and gene-specific effect on longevity associated candidate gene expression in liver**

mRNA levels of several candidate longevity-associated candidate genes – (a) *Parp16*, (b) *Hes6*, (c) *Alas2* and (d) *Hsd3β5*- was assayed in the liver of mice (n=3 per time point) subjected to the following feeding regimens: ad libitum (AL) – blue circles, solid line; 30% calorie restriction (CR) – red squares and solid lines; time restricted feeding (TR)– orange triangles and solid lines, Fasting (F) – green cross and solid lines . For convenience all data are double plotted. Data represents mean ± SD; statistically significant (p<0.05) effects of the feeding (analyzed by two ways ANOVA) at a given time point are indicated by: **a-** between AL and CR groups, **b** – AL and TR, **c**- AL and Fasting, **d-** CR and TR, **e** – CR and Fasting, **f**- TR and Fasting. Light and dark bars at the bottom represent light and dark phase of the day. ZT0 is the time when light is on and ZT12 is the time when light is off. Food for CR and TR groups was provided at ZT14.

**Supplementary Table S1**

| **Primers** | **Forward** | **Reverse** |
| --- | --- | --- |
| ***Bmal1*** | **5’- CAC TGT CCC AGG CAT TCC A- 3’** | **5’- TTC CTC CGC GAT CAT TCG- 3’** |
| ***Clock*** | **5’- GCG ACA GCA GGG ACA CGC CA- 3’** | **5’- CGC GGC GGT AGC GGT GAA TTT T- 3’** |
| ***Per1*** | **5’- AGG TGG CTT TCG TGT TGG-3’** | **5’- CAA TCG ATG GAT CTG CTC TGA G-3’** |
| ***Per2*** | **5’- AAT CTT CCA ACA CTC ACC CC- 3’** | **5’- CCT TCA GGG TCC TTA TCA GTTC-3’** |
| ***Per3*** | **5’- GGT CGA CAT AAA GTC CGA ACG A- 3’** | **5’- TCG TTA CTG GCT GCC TTT TTT ATT- 3’** |
| ***Cry1*** | **5’- CGT CTG TTT GTG ATT CGG GG- 3’** | **5’- ATT CAC GCC ACA GGA GTT GC- 3’** |
| ***Cry2*** | **5’- GGC AGA CCG AGA CCC AGT CCA- 3’** | **5’- ATC GAT TGC GCG GGG ACC G- 3’** |
| ***Rev Erb α*** | **5’- TGG CAT GGT GCT ACT GTG TAA GG- 3’** | **5’- ATA TTC TGT TGG ATG CTC CGG CG- 3’** |
| ***Rev Erb β*** | **5’- GGA GTT CAT GCT TGT GAA GGC TGT- 3’** | **5’- CAG ACA CTT CTT AAA GCG GCA CTG- 3’** |
| ***Ror α*** | **5’- GGA ATC CAT TAT GGT GTC ATT ACG- 3’** | **5’- GTG GCA TTG CTC TGC TGA CTT- 3’** |
| ***Ror γ*** | **5’- ACT ACG GGG TTA TCA CCT GTG AG- 3’** | **5’- GTG CAG GAG TAG GCC ACA TTA C- 3’** |
| ***E4bp4*** | **5’- ACG GAC CAG GGA GCA GAA C- 3’** | **5’- GGA CTT CAG CCT CTC ATC CAT C- 3’** |
| ***Dbp*** | **5’- CCT GAG GAA CAG AAG GAT GA- 3’** | **5’- ATC TGG TTC TCC TTG AGT CTT- 3’** |
| ***Dec1*** | **5’- GCA AGG AAA CTT ACA AAC TGC C- 3’** | **5’- CAA TGC ACT CGT TAA TCC GGT- 3’** |
| ***Dec2*** | **5’- ATT GCT TTA CAG AAT GGG GAG CG- 3’** | **5’- AAA GCG CGC GAG GTA TTG CAA GAC- 3’** |
| ***Tef*** | **5’- GGT CCT GAA GAA GCT GAT- 3’** | **5’- CGT CTT CCT CCG GCT TT- 3’** |
| ***Hlf*** | **5’- CAT CCT GAA GAC GCA TTT A- 3’** | **5’- ATA AGG TGG GTC CCA AG- 3’** |
| ***Ppar α*** | **5’- CCA CCA TCA CTG TAT CT- 3’** | **5’- CAG GAC CTA CTC TCT ATG- 3’** |
| ***18S rRNA*** | **5’- GCT TAA TTT GAC TCA ACA CGG GA- 3’** | **5’- AGC TAT CAA TCT GTC AAT CCT GTC- 3’** |
| ***Gapdh*** | **5’- AAC AGC AAC TCC CAC TCT TC- 3’** | **5’- TGG GTG CAG CGA ACT TTA T- 3’** |

**Supplementary Table S2**

| **Primers** | **Forward** | **Reverse** |
| --- | --- | --- |
| ***Fmo3*** | **5’ CACCACCATCCAGACAGATTAC 3’** | **5’ CCTTGAGAAACAGCCATAGGAG 3’** |
| ***Hsd3β5*** | **5’ GTCAATCTGAAAGGTACTC 3’** | **5’ CCTTGTAGGAATTTGGTC 3’** |
| ***Hes6*** | **5’ CTGTGTTCTGACCTAGAG 3’** | **5’ CTGCCTAAGGATGTAGAC 3’** |
| ***Serpina12*** | **5’ ACCGTGATGATTCTCACAAA 3’** | **5’ AACATCATGGGTACCTTCAC 3’** |
| ***Alas2*** | **5’ GTCTTTGGTTCGTCCTCAG 3’** | **5’ GAACTGGTAGGTTTTAGCCA 3’** |
| ***Igfals*** | **5’ GCTCTGAGAACAGGAAG 3’** | **5’ GATGCTCCAGGATCTGT 3’** |
| ***Parp16*** | **5’ CATAGCCTTCTTGGCCCGAT 3’** | **5’ GCGGTCTATTTCCTTGGAATCCTT 3’** |
| ***Cyp4a14*** | **5’ ACGAGCACACAGATGGAGTG 3’** | **5’ TCTTCTTCCTGGCCTTCTGC 3’** |
| ***Cyp4a12b*** | **5’ CTGATGGACGTTCTTTAC 3’** | **5’ TCAAACACCTCTGGATT 3’** |
| ***Mup4*** | **5’ ACCAAAACCAATCGCTGCCT 3’** | **5’ GCTGTATCGATCGGAAGAGAGG 3’** |

**Supplementary Fig. S1**

**Supplementary Fig. S2**

**Supplementary Fig. S3**
